# Do psychological interventions in childhood and adolescence prevent later mental illness? A commentary on Vassard et al. (2026)

**DOI:** 10.1111/jcpp.70206

**Published:** 2026-07-28

**Authors:** Ian Kelleher

**Affiliations:** ^1^ Centre for Clinical Brain Sciences University of Edinburgh Edinburgh UK; ^2^ School of Medicine University College Dublin Dublin Ireland; ^3^ Faculty of Medicine University of Oulu Oulu Finland; ^4^ St John of God Hospitaller Services Group Stillorgan Ireland

**Keywords:** Early intervention, prevention

## Abstract

Early intervention in youth mental health has long been assumed to confer long‐term benefits, altering developmental trajectories and reducing risk of later mental disorders. The 3‐year follow‐up of the Mind My Mind (MMM) trial challenges these assumptions. The MMM transdiagnostic cognitive behavioural therapy (CBT) intervention led to clinically meaningful improvements at the end of treatment and at short‐term follow‐up. Its superiority over management as usual, however, was not sustained at 3 years, and rates of subsequent specialist psychiatric diagnosis did not differ between the MMM and control groups. These findings highlight that effective psychological intervention in youth does not necessarily alter longer‐term mental health trajectories. Prevention science in youth mental health must move beyond equating early treatment with durable change and instead clarify the mechanisms and targets required to alter developmental trajectories toward serious mental illness.

Child guidance clinics, which emerged more than a century ago, were founded on an ambitious idea: psychosocial interventions in childhood could alter long‐term developmental trajectories and prevent later delinquency and mental ill health. This ambition has remained central to the child and adolescent mental health service (CAMHS) model that emerged from the child guidance movement, as well as influencing subsequent early intervention and stepped‐care models across the adolescent‐to‐adulthood age range. This is now commonly referred to as the ‘intervention as prevention’ model, suggesting that effective early treatment of mental health problems in youth will reduce the risk of later mental illness.

Despite this long history, the long‐term effects of childhood psychological interventions have received relatively little empirical attention. Most psychotherapeutic interventions for children and adolescents have been evaluated at the end of treatment or within a few months of completion (Rith‐Najarian et al., 2019). Evidence of efficacy at these early time points has often been assumed to translate into longer‐term benefit; yet direct empirical tests of this assumption remain scarce.

A meta‐analysis of 110 CBT trials in children and adolescents reported ‘initial support for the durability of treatment effects for youth CBT’. The authors identified five trials reporting on long‐term (2 year+) outcomes for anxiety and two for depression. Only two of these studies, however, had long‐term follow‐up data for individuals in the control arm as well as individuals in the intervention arm. Without a control arm for comparison, long‐term symptomatic improvements in the intervention arms cannot be separated from potential regression to the mean, natural recovery or other non‐specific factors.

In the two studies that followed both the intervention and control arms, there were mixed findings. One of the two trials, which had a minimal intervention control, did not find a sustained benefit of CBT at 2‐year follow‐up (Vostanis, Feehan, & Grattan, 1998). The other trial had a ‘no intervention’ (monitoring) comparison arm and did find a benefit at 2 years (Dadds et al., 1999). However, a ‘no intervention’ control arm introduces a nocebo‐like risk: parents knew their child had anxiety problems but had been randomised to receive no active help. This approach is known to risk biasing results in favour of the intervention arm. Overall, despite prevalent assumptions that early intervention also acts as prevention, there is little empirical evidence to support this hypothesis.

In this context, the 3‐year follow‐up of the Mind My Mind (MMM) trial represents a landmark study on long‐term outcomes of psychological interventions in children and adolescents (Vassard et al., 2026). The original MMM trial randomised nearly 400 help‐seeking 6‐ to 16‐year‐olds with emotional and behavioural problems to a modular transdiagnostic CBT intervention or community‐based management as usual (Jeppesen et al., 2021). The management as usual (MAU) arm provided standardised community‐based care coordination, rather than a specific intervention. Parents received two sessions with a local professional to review their child's needs and help coordinate support from existing municipal services, which ranged widely from no intervention to counselling or school support. This model intentionally lacked specialised therapeutic training and reflected the typical, highly variable level of care available in community settings.

Unlike most previous trials, MMM was explicitly designed for rigorous long‐term evaluation from the start. It involved a pre‐registered protocol, a transdiagnostic modular CBT intervention and patient‐centred and parent‐reported outcomes. It achieved both good questionnaire response rates and 100% registry‐based follow‐up for identifying subsequent presentation to specialist mental health services. Its design, furthermore, included multiple follow‐up time points, allowing us to compare its short‐term effectiveness with other studies and then evaluate how these effects translated, or failed to translate, into longer‐term benefits.

The short‐term outcomes for MMM demonstrated clear, clinically meaningful benefits over MAU on the primary outcome, functional improvement, with effect sizes comparable to those seen in other high‐quality youth psychotherapy trials (Jeppesen et al., 2021). It also demonstrated benefits on a range of secondary outcomes, including improvement in parent‐reported youth anxiety, depressive symptoms, daily and social functioning, school attendance and teacher‐reported impact of problems. These benefits were largely sustained 2 months after the end of treatment. The evidence, overall, was strongly supportive of the clinical benefit of MMM.

The 3‐year follow‐up, however, told a different story. In terms of the primary outcome, functional impairment, the authors found that the initial superiority of the CBT intervention dissipated over time and by the 3‐year follow‐up there was no significant difference between the CBT and control arms. Neither were there substantial differences between the CBT and comparison groups on any parent‐reported secondary outcomes, such as total difficulties, school attendance or parental stress.

What is more, in a concrete test of whether an (effective) psychological intervention in childhood would prevent the subsequent development of mental ill health, the authors found no difference between the CBT and control arms in the number of young people who went on to be diagnosed with mental disorders in specialist mental health services: in both arms, 25% of individuals received a psychiatric diagnosis during the 3‐year follow‐up period.

An additional analysis showed that CBT yielded an incremental gain of 0.121 quality‐adjusted life years (QALY) compared to management as usual during the follow‐up period. This reflects the fact that patients in the CBT arm had faster improvements in their mental health, in line with the original RCT results (Jeppesen et al., 2021). This, of course, is an important outcome in its own right and, with a cost of €24,789 per QALY gained, represents an acceptable cost‐effectiveness ratio.

Altogether, the MMM trial findings appear, at first glance, to represent a paradox. As an intervention to reduce mental ill health in children and adolescents, it was a clear success (Jeppesen et al., 2021). Yet by 3‐year follow‐up, the MMM participants were doing no better than the control group (Vassard et al., 2026). The multi‐stage follow‐up of this well‐designed trial, however, has a crucial lesson to teach us: it is a mistake to assume that effective psychological interventions in childhood and adolescence will necessarily provide long‐term benefits to mental health.

That lesson is, at once, both simple and profound, with important implications for the field. It should not be necessary for mental health treatments in childhood and adolescence to provide benefits years into the future in order to be clinically justifiable – a young person's suffering in the here‐and‐now is worthy of treatment. Nonetheless, it is humbling that assumptions many of us have held about the long‐term benefits of childhood interventions are now in question.

Beyond the need for some humble reflection, these findings provide us with important new challenges – if (effective) psychological interventions do not necessarily provide long‐term benefit, we need to think more ambitiously and radically about prevention. The authors suggest that what may be needed is more sustained psychological intervention. It may be the case that top‐ups would improve longer‐term outcomes. We must not, however, fall into the trap of simply assuming that more‐of‐the‐same will lead to long‐term benefit – if this study teaches us anything, it is that this may not be true and would certainly require empirical testing. It may simply be the case that CBT targets proximal expressions of mental ill health but is less effective in targeting distal risk architectures that affect long‐term trajectories. In that case, more of the same will not lead to longer‐term benefit.

The lack of preventative effect of treatment of anxiety and depression is not limited to CBT; similarly, recent research has suggested that pharmacological treatment of anxiety and depression in childhood and adolescence also does not reduce the risk of later mental illness, at least not for severe mental disorders, such as schizophrenia and bipolar disorder (Healy et al., 2025). As with psychosocial interventions, it may be that pharmacotherapies aimed at prevention need different targets to pharmacotherapies aimed at treatment of established mental illness. This might include targeting, for example, brain plasticity, sleep regulation, stress physiology and infection and inflammation (Lång et al., 2026).

The common thread across these findings is that treatments that (effectively) alleviate acute symptoms do not necessarily alter long‐term trajectories. Prevention and acute treatment, rather, may be fundamentally different enterprises.

A case in point is a recent long‐term follow‐up of an RCT in children and adolescents with parental bereavement. Young people were allocated to either a Family Bereavement Programme (FBP) or a bibliotherapy comparison arm (Sandler, Tein, Zhang, & Wolchik, 2023). The FBP was a 12‐session intervention that targeted caregiver parenting practices and children's coping and grief processing as mechanisms for long‐term prevention. At 15‐year follow‐up, the authors found that young people randomised to FBP had, compared to the control group, approximately half the risk of major depressive disorder and one‐third the risk of generalised anxiety disorder. While such striking results require replication, these findings demonstrate that it may be possible for a relatively brief psychosocial intervention to have meaningful long‐term preventative effects.

Symptom‐focused interventions, whether psychological or pharmacological, are typically designed to reduce current distress and impairment. Prevention, by contrast, may require intervening on the underlying liability structures that shape long‐term risk, such as family dysfunction, educational disengagement, sleep disruption or inflammatory and neurodevelopmental processes. These risk architectures are embedded in social and biological systems. If the underlying architectures remain unchanged, short‐term symptomatic relief may not translate into durable alteration of trajectories. In this sense, the challenge is not simply to intervene earlier or for longer, but to intervene differently.

Ultimately, the MMM long‐term follow‐up challenges us to recognise that prevention in youth mental health cannot be equated with early treatment. Rather than extending existing therapies in the expectation that more‐of‐the‐same will lead to long‐term benefits, we must identify the developmental processes, risk architectures and contextual determinants that truly drive long‐term trajectories toward disorder. Family, school and community‐level strategies, together with individual biological and psychological interventions, should be evaluated with the same rigour exemplified by the MMM trial. And we must heed the lesson that, while early mental health intervention is a clinical imperative in its own right, it is not, by itself, prevention.

## Ethical considerations

Ethics approval is not applicable to this Commentary Article.

## Data Availability

Data sharing not applicable to this article as no datasets were generated or analysed during the current study.
